# Fruit and Vegetable Peels: Utilization of High Value Horticultural Waste in Novel Industrial Applications

**DOI:** 10.3390/molecules25122812

**Published:** 2020-06-18

**Authors:** Harsh Kumar, Kanchan Bhardwaj, Ruchi Sharma, Eugenie Nepovimova, Kamil Kuča, Daljeet Singh Dhanjal, Rachna Verma, Prerna Bhardwaj, Somesh Sharma, Dinesh Kumar

**Affiliations:** 1 School of Bioengineering & Food Technology, Shoolini University of Biotechnology and Management Sciences, Solan-173229, India; microharshs@gmail.com (H.K.); mails4sharmaruchi@gmail.com (R.S.); someshsharma@shooliniuniversity.com (S.S.); 2 School of Biological and Environmental Sciences, Shoolini University of Biotechnology and Management Sciences, Solan-173229, India; kanchankannu1992@gmail.com (K.B.); rachnaverma@shooliniuniversity.com (R.V.); prernabhardwaj135@gmail.com (P.B.); 3 Department of Chemistry, Faculty of Science, University of Hradec Kralove, 50003 Hradec Kralove, Czech Republic; eugenie.nepovimova@uhk.cz; 4 School of Biotechnology and Biosciences, Lovely Professional University, Phagwara-144411, Punjab, India; daljeetdhanjal92@gmail.com

**Keywords:** fruits, vegetables, peels, edible films/coatings, probiotics, nanoparticles, carbon dots, microbiological media, biochar, biosorbents

## Abstract

Fruits and vegetables are the highly used food products amongst the horticultural crops. These items are consumed uncooked, nominally cooked or fully cooked, according to their nature and cooking process. With the change in diet habits and rising population, the production, as well as the processing of horticultural crops, has exponentially improved to meet its increasing demand. A large amount of peel waste is generated from fruit and vegetable-based industries and household kitchen and has led to a big nutritional and economic loss and environmental problems. Processing of fruits and vegetables alone generates a significant waste, which amounts to 25–30% of the total product. Most common wastes include pomace, peels, rind and seeds, which are highly rich in valuable bioactive compounds such as carotenoids, enzymes, polyphenols, oils, vitamins and many other compounds. These bioactive compounds show their application in various industries such as food to develop edible films, food industries for probiotics and other industries for valuable products. The utilization of these low-cost waste horticultural wastes for producing the value-added product is a novel step in its sustainable utilization. The present review intends to summarize the different types of waste originating from fruits as well as vegetables peels and highlight their potential in developing edible films, probiotics, nanoparticles, carbon dots, microbial media, biochar and biosorbents.

## 1. Introduction

In the European Union, approximately 89 million tons of food waste is generated, and this value is expected to increase by 40-fold in coming years. The Food and Agriculture Organization (FAO) estimated that around 40% of the food produced in India is wasted [1,2]. Moreover, the Food Corporation of India reported this loss ranging between 10 to 15 percent of the total production. The Ministry of Food Processing Industries (MFPI) India estimated fruit and vegetable losses to be 12 and 21 million tons, respectively, amounting to an approximate value of about 4.4 billion USD, with a total food value loss and waste produce of 10.6 billion USD [2]. A broader term is “fruit and vegetable waste” (FVW), which refers to indigestible parts that are thrown away at different stages such as collection, handling, shipping and processing [3]. In accordance with the above definition, FVW can be considered as fruit and vegetable loss instead of waste. FVW can be produced at different steps from farm to consumer, involving both pre- and post-consumer stages of the food supply chain [4].

**Figure 1:**
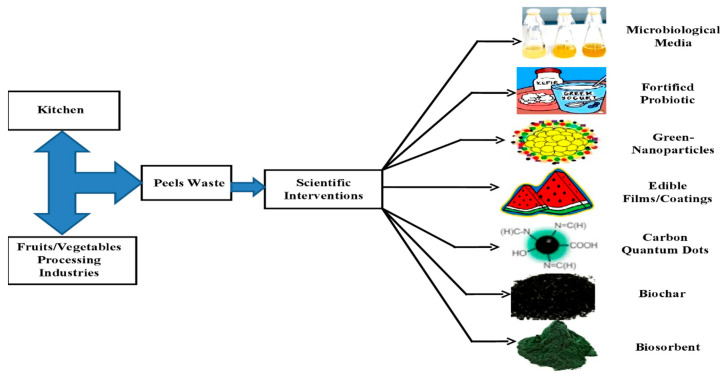
Utilization of fruits and vegetable peel-based waste into novel industrial products [10,11,12,13,14,15,16].

There are high amounts of phytochemical constituents present in FVW and studied for dietary fibers, phenolic compounds and other bioactive compounds extraction [5]. Studies revealed that essential nutrients and phytochemicals are abundantly found in the peels, seeds and other constituent of vegetables and fruits in common use [6]. For instance, the skin of avocados, grapes, lemons, seeds of jackfruits and mangoes contains 15% higher phenolic concentrations as compared to fruit pulp [7,8]. The FVWs can be utilized to extract as well as obtain bioactive compounds that can be used in cosmetics, food, textile and pharmaceutical industries as shown in Figure 1. Some of the FVWs waste originates from horticultural supplies, which is not considered important at present. Their proper use will not only solve the environmental issues, but will act as a sustainable approach to improve health via enriched food containing health-enhancing substances [9]. The current review summarizes the scientific inventions and reviews the recent advances in the exploitation of waste fruits and vegetables peel as a valuable commodity of the future.

## 2. Fruit and Vegetable Peel Based Edible Coatings/Films

Edible coatings are made up of thin layers applied on the surface of the food to make its shelf life longer, maintain the properties, characteristics and functionality of foods at minimum cost [17]. This application can increase its functionality by extending the shelf life, preventing the microbial spoilage and acting as a carrier matrix for antimicrobial agents [18,19]. Coating can be considered as an effective method of preservation during transport of fruits and vegetables easily affected by microorganisms, insects, pre- and post-harvesting conditions [20]. Coatings also help in developing a modified atmosphere to induce varied alterations in minimally processed and fresh foodstuff in various areas such as sensory quality, antioxidant properties, color, firmness, ethylene production, microbial growth inhibition and organic compounds under anaerobic processes [21]. Recently, essential oils (EOs) and their main components have received substantial attention due to the presence of effective antimicrobial properties in them. The main EO component of lemongrass citral (3,7-dimethyl-2,6-octadienal) has been reported for its antimicrobial activity against a variety of foodborne pathogens and is also explored as an antimicrobial agent in edible coatings [22]. The EOs are commonly considered as safe, as they impart maximum effects with the minimum change in the organoleptic properties of the food [23]. Of late, in the development of edible coatings applications of this emerging technology included a variety of nanosystems, consisting of nanoemulsions, polymeric nanoparticles and nanocomposites to release antioxidants, and showing antibacterial activities on the surface of the food. Because of the many phenolic substances with excellent antioxidant capacity, fruits and vegetable peel are considered as suitable materials for inclusion into films and coatings.

Fish gelatin is believed to be a valuable biopolymer source for fabricating biofilms because of its biodegradable nature and high myofibrillar protein content [24]. Additionally, because of the variations in the sequence of amino acid, fruit and vegetable peel-based films show less water permeability in contrast to mammalian gelatin-based film. Enriching pomegranate peel powder in gelatin films considerably increased their water vapor permeability (WVP) as incomplete dissolution of pomegranate peel in the film matrix resulted in more heterogeneous microstructure [25]. Both hydrophobic and hydrophilic components present in the peel of pomegranate balances the hygroscopic properties and do not alter the moisture content of the films (Table 1).

The peel of potato contains adequate quantities of cellulose, fermentable sugars, hemicellulose, and starch [33]. The films with low concentration peel of potato after comparison with high concentrations peel in the biopolymer film resulted in a higher WVP due to bigger pore size of film matrix despite its denser structure [33]. Potato peel biopolymer film-coating proved helpful for designing biodegradable food packaging with more-value commercial use. Generally, fish gelatin/polyethylene bilayer films solubility was lowered by enriching with different fruit peels [34].

## 3. Fruit and Vegetable Peel Fortified Probiotics

Over the past two centuries, fruits have been used as a remedy for dry cough, severe thirst and sore throat in medicine. In the last few years, the demand for novel functional foods has increased and probiotics are commonly consumed all over the world and considered as one of the main functional food products [35]. Additionally, the fruits and peels are of great value and high source of bioactive compounds. In pomegranate, citrus, mango and *Opuntia ficus-indica* (barbary fig) peel, the functional ingredients present in abundance are antioxidants, fiber and oligosaccharides (as prebiotics) [36,37,38,39]. Probiotics and dietary fiber both have been reported to reduce the incidence of colon cancer and relieve constipation [40]. Additionally, some dietetic fibers obtained from fruits have shown considerable effect on the viability of these bacteria and are recommended as an ingredient in probiotic dairy foods [11]. Various attempts have been made to increase the biological activities of probiotics, including supplementation with fruit peel.

Probiotic yogurt prepared with pineapple peel powder improved the anticancer, antioxidant and antibacterial activities against *Escherichia coli*, but no significant effect was observed on *Staphylococcus aureus* [41]. The addition of apple, banana and passion fruit peel powder in probiotic yogurt improved the rheological properties and enhanced the growth of *Lactobacillus casei*, *Bifidobacterium animalis* subsp. *lactis*, *Lactobacillus acidophilus* and *Lactobacillus paracasei* [11]. The effect of milk supplementation with mango peels on the kefir microorganism’s growth rates and antioxidant properties were also estimated in fermented products [42]. Composite fruit peel powder (orange, passion fruit and pineapple) was used in different proportions i.e., 1%, 0.5% and 0.7% (*w*/*v*), respectively, to develop fat and sugar-free probiotic set yogurt [43]. Increase in firmness and consumer acceptability, decrease in syneresis and high lactic acid bacteria counts were observed in yogurt incorporated with 0.5% peel mixtures.

## 4. Fruit and Vegetable Peel-Derived Metallic Nanoparticles

Beneficial bioactive molecules such as alkaloids, amino acids, enzymes, phenolics, proteins, polysaccharides, tannins, saponins, vitamins and terpenoids and other compounds present in fruits and vegetable waste generally act as reducing agents in metal nanoparticles (NPs) synthesis [44,45]. Some biomolecules play the role of modeling agents directing particle growth in a specific direction, while other biomolecules function as capping agents, preventing nanoparticle from getting agglomerated [46,47]. Nanoparticles biosynthesized by using FVW have also emerged as a reliable, sustainable and eco-friendly technology with lower risk to human health and environmental as compared with chemicals and toxic solvents based conventional manufacturing protocol [48]. A significant interest in use of NPs was reported due to their distinctive physicochemical properties and applications in various fields of biomedicine and pharmaceuticals. The biogenic NPs are synthesized by following the bottom-up approach, in which atoms as well as compounds act as the building block and self-assembled themselves to form the final product [49,50].

Various noble metal/metal oxide NPs have been synthesized from fruits and vegetables peel extracts as shown in Table 2.

Gold (Au) NPs have been synthesized using dried onion peels (OP) aqueous extract, which reduces Au^3+^ to OP-AuNPs by forming a colloidal solution [64]. The phytoconstituents, mainly the cysteine derivatives, found in the onion bulb, might be responsible for the synthesis of OP-AuNPs. Zinc oxide (ZnO) nanoparticles were synthesized with domestic waste potato peel after 24 h of incubation using starch present in the potato peel to reduce the metal ion [63]. Banana, pomegranate, lemon and orange peel extracts have also been found to possess the ability to reduce silver (Ag^+^) ions in aqueous solutions to synthesize silver (Ag) nanoparticles [54,55,56,57]. Likewise, ZnO-NPs have been synthesized using tomato, grapefruit, lemon and orange peel [61].

## 5. Fruit and Vegetable Peel Derived Carbon Dots

Carbon dots (CDs) are a very small (<10 nm) photoluminescent material synthesized by two approaches, i.e., top-down and bottom-up synthetic routes [67,68]. In the top-down synthetic route, a large carbon structure breaks down by involving acid assisted chemical oxidation, electro-oxidation or laser ablation in the synthesis process [69]. However, this approach requires a complex and extreme synthetic condition, which is considered as one of the disadvantages of this method. On the other hand, the bottom-up approach using plants and their byproducts without using chemicals was found advantageous over the top-down approach. Food waste is a matter of serious concern worldwide, and this waste needs to be given proper attention. The use of food as waste offer economic benefits and is one of the most interesting starting materials as carbon sources for the synthesis of CDs [67]. Presence of functional components such as carotenoids, dietary fiber, gallic acid, polyphenols, flavonoids and mangiferin in mango peel and pineapple peel makes these suitable for the development of CDs [70,71]. The increasing number of publications shows that the fruit and vegetable by-products are source of antibacterial, antioxidant and nutritional dietary fiber [72]. Additionally, because of their biocompatibility, innocuousness, low toxicity, low cost and photostability properties, they are suitable starting materials for CDs [73,74]. The appropriate properties, i.e., pyrolyzation at high temperature and oxygenolysis with concentrated acid, carbonization, oxidation, polymerization and nucleation are needed during peels treatment to synthesize CDs [14]. CDs have shown a promising potential for applications in biomedical to energy storage devices, determination of pathogens, environmental studies, detection of heavy metals and additives in the food and water purification processes (Table 3) [75,76].

## 6. Fruit and Vegetable Peel Based Microbiological Media

In microbiological studies, microorganisms are grown by adding suitable culture media with favorable environment under laboratory conditions [89]. In most of the cases, commercially available media such as Cetrimide agar, Nutrient agar and MacConkey agar are used, but these are generally considered high cost media [89]. The growth and isolation of organisms have been reported using different substrates and media [90]. Some fruits and vegetables, such as cabbage, carrot, gooseberry, tomato, pumpkin etc., have been used as a substitute for nutrient agar to culture both bacteria and fungi, [91]. Other reports have used black gram, cowpea and green gram as starch and protein substitutes to ease the microbial media cost [92]. Fruits and vegetable bio-waste contain simple and complex sugars that are metabolized by microorganisms and have received much attention for their use in animal feed, bio-ethanol and biogas production [93,94].

Different type of agriculture waste is now used for the production of low-cost growth media for microorganisms (Table 4) [95,96].

Agar of Dragon fruit peel (DFP) was also used as a microbial growth media [92]. Grapefruit, banana and melon peels contain a high amount of carbohydrates, which act as a good substrate for the production of amylase [10]. Banana peel was found to be an economically low and effective medium for the growth of fungi [98]. The watermelon peel extract is found rich in macronutrients such as lipids, reducing sugars and total proteins [99]. The study reported that watermelon peel waste (WPW) was best for the growth of *Aspergillus niger*, *Fusarium oxysporum*, *Lichtheimia corymbifera*, *Penicillium expansium* and *Rhizopus oryzae,* respectively. The study also revealed that formulated watermelon peel waste dextrose agar (WPWDA) (watermelon peel waste dextrose agar) medium was found as an alternative way for some commonly used media such as Czapek’s Dox agar (CzDA) and Potato dextrose agar (PDA); in addition, this medium was very cheap and eco-friendly. Orange peel waste (OPW) was used as a liquid medium in producing biodiesel used oleaginous yeasts and found that and *Cryptococcus laurentii* UCD 68–201 and *Rhodosporidium toruloides* NRRL 1091 strains yielded 31.9% and 36.9% of biodiesel, respectively [100]. Pea peel waste was used as a growth medium at 30 °C with *Trichoderma reesei* to produce cellulase enzyme [102].

## 7. Fruit and Vegetable Peel Derived Biochar

Biochar is a stable carbon-rich solid generated by pyrolysis as a result of the thermochemical decomposition of organic feedstock material at high temperatures under oxygen-free conditions [104]. Different types of food waste have been used for the production of biochar and its yield and physicochemical properties have been reported in detail [105,106]. Biochar is generally used to remove different types of pollutant containing heavy metals from contaminated water bodies [107,108,109]. It also serves as an intermediate for producing bioethanol from biological waste collected from food processing industries as well as different agricultural plant residues like husk, bran etc. [110,111]. Different studies have shown the production of biochar from different types of fruits and vegetables peel wastes (Table 5).

Potato peel waste (PPW) was used to produce biochar by fast pyrolysis using the fluidized bed system to remove H_2_S [122]. Biochar produced from pineapple peel showed that H-bonding interacts with oxytetracycline (OTC) for its sorption. However, thermodynamic parameters showed that the OTC sorption onto the biochar was endothermic and is a spontaneous process [115]. Biochar derived from pineapple, sweet lime and pomelo peel was developed to remove hexavalent chromium from aqueous solution [114,117,121]. In another report, biochar was prepared from rambutan and pomegranate peel for the removal of copper (II) ions from aqueous and soil system, respectively [118,119]. Biochar derived from pomelo and litchi peels were used to remove congo red, methyl orange and malachite green from wastewater [113,118].

## 8. Fruit and Vegetable Peel Derived Biosorbents

Biosorption can be explained as a mechanism when a sorbate (i.e., an atom, ion or compound) reacts with the biomass or biomaterial (stated as biosorbent), which causes the acclimatization of sorbate ions over the surface of biosorbents, which subsequently reduces the sorbate concentration in the solution [123]. This mechanism has attained significant attention due to its ability to immobilize the heavy metal contaminated from the water (especially contaminated with the discharge of electroplating and mining industries) or metal processing industries. Numerous biosorbents have been developed with the help of different biomasses such as algae, fungi (e.g., *Mucorrouxii*), yeasts and bacteria (e.g., *Bacillus thuringiensis*) [124,125]. The natural biomass complex compendium signifies the contribution of different processes that describe the mechanism of how biosorbents works in eliminating the various contaminants; however, these processes are still being explored. Several functional groups are attached to these biosorbents to attract and sequester the contaminants, which relies on the type of biosorbent and functional groups (amine, amides carboxyl, hydroxyl, carbonyl, sulfhydryl, phenolic, sulfonate and phosphate groups) attached to it [126,127].

Many studies were done to produce biosorbents from fruits peel, i.e., apple, pineapple and dragon fruit and vegetables peel such as garlic, and cucumber to remove methylene blue dye from the aqueous solution (Table 6) [16,128,129,130,131].

Sponge gourd peel was considered as an inexpensive natural biosorbent to remove malachite green (MG), a cationic dye, in batch mode [140]. Banana peel was used as an efficient biosorbent for removing rhodamine-B, a cationic water-soluble dye of basic nature [138]. The contact time selected for the adsorption of rhodamine-B on banana peel powder was 60 min. In another study, a bisorbent was developed by using natural banana peel (NBP), methylated banana peel (MBP) and banana peel activated carbon (BPAC), respectively, and used in the treatment of palm oil mill effluent (POME) [136].

## 9. Conclusions

Development of the sustainable solution for managing fruit and vegetable waste has become extremely important in present scenario. Therefore, it demands the development of solution that could utilize the full potential of these waste material and support in attaining the social, environmental and economic benefits from these wastes. Furthermore, utilization of the fruit as well as vegetable waste especially peels in developing value-added products such as edible films, probiotics, nanoparticles, carbon dots, biochar and biosorbents will be an eco-friendly and sustainable way to create novel business opportunities and also functionalizing this waste for a useful purpose. Most of these interventions are in its infancy stage and lacks the technological advances and findings. Hence, there is high need to develop consortia of researcher and industrialist to improve the economic potential of these valuable horticultural wastes with a support of initial investment. Moreover, it will aid in promoting the usage of horticultural waste for synthesizing value-added commodities.

## Figures and Tables

**Table 1:** Fruits and vegetables peel based edible films/coating with their applications.

Fruit/Vegetable Common Name	Scientific Name	Matrix	Applied on Food Items	Technique Used	Beneficial Effects	Ref
Apple	*Malus domestica*	Carboxy methylcellulose	Fresh beef patties	Microfluidization	A complete inhibition of lipid oxidation, and efficient suppression of the growth of microbes on raw beef patties. No effect on the sensory characteristics of raw and cooked beef patties	[26]
Orange	*Citrus sinensis*	Gelatin	Cupcake	ND	Increase in peroxide value by 3.60–4.80 (mL.eq./kg fat) in refrigerated storage for 1 week and decrease in microbial growth	[27]
Pomegranate	*Punica granatum*	Mung bean protein	NS	ND	The films enriched with pomegranate peel also showed higher total phenolic content; antioxidant activity, antibacterial capacity compared to the control mung bean protein film. These films found their use in food industry to develop bio-functional edible films intended for packaging of food products	[28]
Potato	*Solanum tuberosum*	Oregano essential oil (OEO)	Cold-smoked salmon	ND	When samples were coated with Potato processing waste-based-oregano oil-incorporating film (PPW-OO), the *Listeria* population decreased from 6.7 to 4.7 log CFU/g by the end of storage. Incorporation of oil into the films reduced the film strength and increased their water vapor permeability. The PPW-OO film reduced the growth of *Listeria monocytogenes* on cold-smoked salmon during storage under vacuum conditions at 4 °C for 28 days	[29]
Orange	*Citrus sinensis* (L.) Osbeck	Chitosan film	Deepwater pink shrimp	Casting	The combination of chitosan film with 2% orange peel essential oil concentration was effective in prolonging the shelf life of fresh shrimps to 15 days	[30]
Orange	*Citrus sinensis* (L.) Osbeck	Gelatin	Shrimps	ND	Gelatin coating combined with orange peel essential oil preserved shrimp quality during cold storage with a shelf-life extension of about 6 days	[23]
Lemon	*Citrus limon*	Cassava starch and sodium alginate	Tofu, Strawberry	ND	The addition of 0.6% lemon peel essential oil (LPEO) to tofu and 1% LPEO to strawberry with each of edible coating agents was significantly able to reduce their degradation	[31]
Orange	*Citrus sinensis*	Carnauba wax, montmorillonite nanoclay	Blood orange	ND	Blood orange coated by carnauba wax with montmorillonite nanoclay (MMT) had the least deformation and dissolved solid and the highest acidity compared to other treatments. Fruits coating with MMT showed better brightness	[13]
Orange	*Citrus sinensis*	Pectin-coating	Fresh-cut orange	ND	The results showed that the nanoemulsion-based edible coatings containing orange peel essential oil can extend the shelf life of orange slices without any undesirable impacts on sensory attributes	[32]

NS: not specified; ND: not defined.

**Table 2:** Fruits and vegetables peel derived metallic nanoparticles with their application.

Fruit/Vegetable Common Name	Scientific Name	Types of Nanoparticles Synthesized	Reaction Time	Morphology	Size	Applications	Ref
Pomegranate; Orange; Banana and Apple	*Punica granatum*; *Citrus sinensis*; *Musa*; *Malus domestica*	Silver	2 min	Sphere	25 nm	Antibacterial activity against *Salmonella* sp., *Escherichia coli*, *Pseudomonas* sp., *Aeromonas hydrophila;* Antifungal activity against *Fuarium* sp.; Antioxidant activity using 2,2-diphenyl-1-picrylhydrazyl (DPPH); Cytotoxicity against human breast cancer cells MCF-7	[51]
Orange; Banana	*Citrus sinensis*; *Musa*	Silver	1 h	Sphere	ND	Antibacterial activity against *Staphylococcus aureus*, *Proteus vulgaris*	[52]
Orange; Lemon; Sweet lemon	*Citrus sinensis*; *Citrus limon*; *Citrus limetta*	Silver	24 h	ND	ND	Antibacterial activity against *Pseudomonas aeruginosa*, *E. coli* and *Salmonella typhimurium*	[53]
Orange	*Citrus sinensis*	Silver	10 min	Sphere	47–53 nm	Photocatalytic against methylene blue	[54]
Lemon	*Citrus limon*	Silver	30 min	Sphere	2–5 nm	Antibacterial activity against *P. aeruginosa*, *E. coli*, *Acinetobacter baumannii*, *Streptococcus mutans*, *Proteus mirabilis*; Antifungal activity against *Candida albicans*	[55]
Pomegranate	*Punica granatum*	Silver	24 h	ND	5–50 nm	Antibacterial activity against *S. aureus*, *P. aeruginosa*, *E. coli*	[12]
Banana	*Musa paradisiaca*	Silver	1 h	Sphere	23.7 nm	Antibacterial activity against *P. aeruginosa*, *E. coli*, *S. aureus*, *Bacillus subtilis*; Antifungal activity against *C. albicans*	[56]
Pomegranate	*Punica granatum*	Silver	24 h	Sphere	20–40 nm	Antibacterial activity against *E. coli*, *P. vulgaris*, *P. aeruginosa*, *S. typhimurium*, *S. aureus*, *Staphylococcus epidermidis*, *Klebsiella pneumonia*; Cytotoxicity against human colon cancer cell line RKO: ATCC^®^ CRL-2577™	[57]
Apricot	*Prunusa rmeniaca*	Silver	NS	Rod	50 nm	Antibacterial activity against *E. coli*, *S. aureus*, *P. aeruginosa*, *B. subtilis*	[58]
Cavendish banana	*Musa acuminata*	Silver	30 min	Sphere	55 nm	Antibacterial activity against *S. aureus*, *B. subtilis*, *E. coli*, *K. pneumonia*; Antioxidant activity (DPPH), 2,2’-azino-bis(3-ethylbenzothiazoline-6-sulfonic acid) (ABTS)	[59]
Orange	*Citrus sinensis*	Silver	5 h	ND	48.1 nm	Antibacterial activity against *Xanthomonas axonopodis* pv. *citri* (*Xac*)	[60]
Tomato; Orange; Grapefruit; Lemon	*Lycopersicon esculentum*; *Citrus sinensis*; *Citrus* *Paradise*; *Citrus aurantifolia*	Zinc Oxide	1 h	Hexagonal	9.01 nm; 12.55 nm; 19.66 nm; 11.39 nm	Photocatalytic against methylene blue	[61]
Sweet Potato	*Ipomoea batatas* (L.) Lam.(Ib)	Silver	1–12 h	Agglomerated	ND	Antibacterial activity against *Enterococcus feacium*, *Salmonella enteritica, Listeria monocytogenes*, *B. cereus*, *S. aureus*; Antidiabetic; Antioxidant activity (DPPH, ABTS, nitrite/nitrate oxide (NOx)); Cytotoxicity against HepG2 cancer cells	[62]
Potato	*Solanum tuberosum*	Zinc Oxide	24 h	Hexagonal	30–150 nm	Photocatalytic against methylene blue and azo dyes	[63]
Onion	*Allium cepa*	Gold	24 h	Sphere and Triangle	45.42 nm	Synergistic antimicrobial potential against *B. cereus*, *E. coli*, *L. monocytogenes*, *S. aureus*, *S. typhimurium*; Antifungal activity against *C. albicans*, *C. glabrata*, *C. glochares*; Antioxidant activity (DPPH, ABTS, NOx)	[64]
Bottle gourd	*Lagenaria siceraria*	Silver	20 h	Sphere	5–40 nm	Cytotoxicity against A431, (skin carcinoma, p53 mutant) and A549, (lung carcinoma, p53 wild type); Antibacterial activity against *S. typhi*	[65]
Radish	*Raphanus sativus*	Silver	15 min	Polygonal	30–60 nm	Antibacterial activity against *S. aureus*, *B. subtilis*, *E. coli*, *K. pneumonia*	[66]

NS: not specified; ND: not defined.

**Table 3:** Fruits and vegetables peel as a carbon source for preparing carbon dots.

Fruits/Vegetable Common Name	Scientific Name	Production Conditions	Detection Limit of Heavy Metals	Applications	Ref
Mango	*Mangifera indica*	Hydrothermal/300 °C/2 h	1.2 µM	Cellular labeling ferrous ion (Fe^2+^) detection	[14]
Pineapple	*Ananas comosus*	Hydrothermal/200 °C/3 h	4.5 nM	Electronic security devices mercury ion (Hg^2+^) quantification	[77]
Lemon	*Citrus limon* (L.)	Hydrothermal/200 °C/8 h	73 nM	Cr^6+^ sensing; Photocatalysis effect	[75]
Sweet lemon	*Citrus limetta*	Hydrothermal/180 °C/3 h	NA	Breast cancer detection gene therapy	[78]
Banana	*Musa acuminata*	Microwave-assisted/500 W/20 min	NA	Determination of colitoxin DNA	[79]
Pomelo	*Citrus maxima*	Hydrothermal/200 °C/3 h	0.23 nM	Hg^2+^ sensing	[80]
Grapefruit	*Citrus paradisi*	Hydrothermal/190 °C/12 h	NA	Photoluminescence immunoassay	[81]
Onion	*Allium cepa*	Microwave-assisted/1000 W/a specific time intervals	NA	Skin wound healing; Living cells imaging	[82]
Watermelon	*Citrullus lanatus*	Hydrothermal/220 °C/2 h	NA	Imaging probe	[83]
Citrus	*Citrus sinensis*, *Citrus limon*	Hydrothermal/180 °C/2 h	0.01 µM	Ferric ion (Fe^3+^) and tartrazine sensing; Cell imaging	[84]
Orange	*Citrus sinensis*	Hydrothermal/150 °C/10 h	NA	Photocatalytic activity	[85]
Mangosteen	*Garcinia mangostana*	Hydrothermal/200 °C/30 min	NA	Cells imaging	[86]
Pomegranate	*Punica granatum*	Hydrothermal/180 °C/36 h	NA	Recovery of latent prints	[87]
Banana	*Musa acuminata*	Hydrothermal/200 °C/2 h	211 nM	Selective and sensitive detection of Fe^3+^ ions	[88]

NA-not applicable.

**Table 4:** Fruits and vegetables peel based microbiological media.

Fruit/Vegetable Common Name	Scientific Name	Medium Composition	Purpose/Utilization	Ref
Dragon fruit	*Hylocereus undatus*	Dragon fruit peel powder (33.3 g/L), peptone (20 mg/mL) and agar (1.5%)	Viability analysis of *Escherichia coli*	[97]
Orange; Potato; Drum stick	*Citrus sinensis*; *Solanum tuberosum*; *Moringa oleifera*	Peel powder of orange (0.20 g/100 mL), potato (0.25 g/100 mL), drum stick (1 g/100mL) and agar (2%)	Growth and pigment production analysis of *E. coli*, *Serratia* sp., *Pseudomonas* sp.	[89]
Banana; Melon; Grapefruit	*Musa*; *Cucumis melo*; *Citrus paradise*	Luria-Bertani medium contained 1% (*w*/*v*) starch, banana, grape fruit and melon peel powder	Amylase production from *Bacillus* sp. AY3	[10]
Banana	*Musa*	Autoclave banana peel directly inoculated with fungi	Growth of human fungal pathogens viz. *Lasiodiplodia theobromae*, *Macrophomina phaseolina*, *Nigrospora sphaerica*, *Chaetomium murorum*, *Nattrassia mangiferae* and *Schizophyllum commune*	[98]
Watermelon	*Citrullus lanatus*	Watermelon peel waste extract (500 g/L) and dextrose (20 g/L)	Evaluation of fungal growth such as *Rhizopus oryzae*, *Lichtheimia corymbifera*, *Aspergillus niger*, *Penicillium Expansium* and *Fusarium oxysporum*	[99]
Orange	*Citrus sinensis*	Orange peel extract (19.8 g/L), (NH_4_)_2_SO_4_ (0.6 g/L)	Biodiesel production using oleaginous yeasts	[100]
Sponge gourd; Lychee	*Luffa cylindrica*; *Litchi chinensis*	Sponge gourd peel bed soaked with urea (0.3 g/L), (NH_4_)_2_SO_4_ (1.4 g/L), KH_2_PO_4_ (2.0 g/L), MgSO_4_ 7H_2_O (0.3 g/L), peptone (1 g/L), tween 80 (0.2 g/L), FeSO_4_ 7H_2_O (0.005 g/L), MnSO_4_.7H_2_O (0.0016 g/L), ZnSO_4_. 7H_2_O (0.0014 g/L) CaCl_2_ 2H_2_O (0.4 g/L), CoCl_2_ 6H_2_O (0.02 g/L); same composition with lychee peel	Cellulase production using *Trichoderma reesei*	[101]
Pea	*Pisum sativum*	Pea peel powder soaked with urea (0.3 g/L), (NH_4_)_2_SO_4_ (1.4 g/L), KH_2_PO_4_ (2.0 g/L), MgSO_4_.7H_2_O (0.3g/L), peptone (1g/L), tween 80 (0.2 g/L), FeSO_4_ 7H_2_O (0.005 g/L), MnSO_4_.7H_2_O (0.0016g/L), ZnSO_4_. 7H_2_O (0.0014 g/L) CaCl_2_.2H_2_O (0.2 g/L), CoCl_2_. 6H_2_O (0.2 g/L)	Cellulase production using *Trichoderma reesei*	[102]
Orange; Potato; Drum stick	*Citrus sinensis*; *Solanum tuberosum*; *Moringa oleifera*	Peel powder of orange (0.20 g/100 mL), potato (0.25 g/100 mL), drum stick (1 g/100 mL) and agar (2.5%)	Growth analysis of *Trichoderma* sp., *Aspergillus* sp.	[103]

**Table 5:** Fruits and vegetables peel derived biochar and its applications.

Fruit/Vegetable Common Name	Scientific Name	Process conditions Required for Biochar Formation	Applications	Ref
Orange; Banana	*Citrus sinensis*; *Musa*	Pyrolysis at 500 °C for 10 min	Showed good performance in reducing the concentration of biochemical oxygen demand (BOD), chemical oxygen demand (COD), total suspended solid (TSS) and oil and grease of Palm oil Mil effluent (POME) to an acceptable level below the discharge	[112]
Banana	*Musa*	Hydrothermal carbonization at 230 °C for 2 h	Showed excellent lead clarification capability of 359 mg/g and 193 mg/g, respectively	[15]
Pomelo	*Citrus maxima*	Pyrolysis at 450 °C for 1 h	One gram of biochar adsorb 150 mg/L methyl orange dye	[113]
Pineapple	*Ananas comosus*	Pyrolysis at 750 °C for 2 h	Sorption capacity for hexavalent chromium: Cr (VI) was 7.44 mg/g	[114]
Pineapple	*Ananas comosus*	Pyrolysis at 200 °C for 2 h and then heated at 650 °C for 3 h	Sorption of oxytetracycline	[115]
Orange; Pineapple; Dragon fruit	*Citrus sinensis*; *Ananas comosus*; *Hylocereus undatus*	Pyrolysis at 300 °C for 2 h	Maximum ammonium cation (NH^4+^) adsorption capacities were associated with biochars of orange peel (4.71 mg/g) and pineapple peel (5.60 mg/g) produced at 300 °C for 2 h. The maximum NH^4+^ adsorption capacity of the dragon fruit (pitaya) peel biochar produced at 400 °C for 2 h was 2.65 mg/g	[116]
Pomelo	*Citrus maxima*	Pyrolysis at 450 °C for 1 h	A 0.05 g of biochar adsorbed 57.637 mg/g of Cr (VI)	[117]
Litchi	*Litchi chinensis*	Hydrothermal carbonization at 180 °C for 12 h	Adsorption capacity for congored and malachite green was 404.4 and 2468 mg/g	[118]
Rambutan	*Nephelium lappaceum*	Pyrolysis at 600 °C for 3 h	Adsorption for removal of copper ion: Cu(II) from aqueous solutions of 50 and 100 mg/L at 0.2 and 0.4 g/L adsorbent dosages, respectively	[119]
Pomegranate	*Punica granatum*	Pyrolysis at 300 °C for 2 h	Adsorption of Cu(II) was 52 mg/g	[120]
Sweet lime	*Citrus limetta*	Pyrolysis at 450 °C for 1 h	Maximum removal efficiency was found to be 95% with 120 mg/L of initial Cr(VI) concentration with 3 g/L of biochar dose	[121]
Potato	*Solanum tuberosum*	Pyrolysis at 500 °C for 5 min	Hydrogen sulfide (H_2_S) was achieved 53 mg/g at 500 °C, under space velocity (8000 L min^–1^kg^–1^)	[122]

**Table 6:** Fruits and vegetables peel derived biosorbents and their applications.

Fruit/Vegetable Common Name	Scientific Name	Drying Temperature/Time	Applications	Ref
Apple	*Malus domestica*	60 °C/24 h	Adsorbed 107.52 mg/g of methylene blue	[16]
Dragon fruit	*Hylocereus undatus*	105 °C/24 h	A dosage of 0.06 g adsorbed 192.31 mg/g of methylene blue	[131]
Pineapple	*Ananas comosus*	70 °C/48 h	Adsorbed 97.09 mg/g of methylene blue	[129]
Grapefruit	*Citrus paradisi*	105 °C/24 h	Adsorbed 52.48 mg/g copper ion: Cu(II)	[132]
Banana	*Musa paradisiaca*	60 °C/5 h	Removed 90% lead (II) and cadmium (II) ions	[133]
Langast	*Lansium domesticum*	60 °C/24 h	Adsorbed 10.1 mg/g of nickel	[134]
Ponkan fruits/Mandarin orange	*Citrus reticulata*	RT/days	Adsorbed 112.1 mg/g of lead (II) ions	[135]
Banana	*Musa*	80 °C/48 h	Adsorbed 97 mg/g color, 25 mg/g TSS, and 90.5 mg/g COD removed from Palm oil mill effluent (Natural banana peel); Adsorbed 137.5 mg/g, 28.5 mg/g and 93 mg/g for color, TSS and COD removed (Methylated banana peel)	[136]
Ponkan fruits/Mandarin orange	*Citrus reticulata*	60 °C/24 h	Adsorbed 1.92, 1.37 and 1.31 mmol/g of nickel (II), cobalt (II) and copper (II) ions	[137]
Banana	*Musa*	RT/4 days	A dosage of 0.3 g adsorbed 81.07% of rhodamine-B	[138]
Bottle gourd	*Lagenaria siceraria*	80 °C/24 h	Adsorbed 99% copper, 95% silver and iron	[139]
Sponge gourd	*Luffa acutangula*	60 °C/24 h	A dosage of 8 g/L adsorbed 69.64 mg/g of malachite green	[140]
Potato; Carrot	*Solanum tuberosum*/ *Daucus carota* subsp. *sativus*	60 °C/48 h	A dosage of 3.0 g adsorbed 79.32% of nickel	[141]
Cucumber	*Cucumis sativus*	95 °C/24 h	A dosage of 4 g/L adsorbed 81.4% methylene blue	[130]
Garlic	*Allium sativum*	60 °C/24 h	Adsorbed 142.86 mg/g of methylene blue	[128]

RT-room temperature.
